# 590. Impact of Universal Nasal Decolonization with an Alcohol-Based Nasal Antiseptic (ABNA) Versus Mupirocin on Infection Rates and Antimicrobial Utilization in Patients (Pts) Admitted to the Burn Service

**DOI:** 10.1093/ofid/ofae631.185

**Published:** 2025-01-29

**Authors:** Arefa Bacchus, Werner Bischoff, Mary Banoub, Olivia Randazza, Vera P Luther, James R Beardsley, John C Williamson, Kristin Rebo, Seth Garner, Michael E DeWitt, Alex D Taylor

**Affiliations:** Atrium Health Wake Forest Baptist, Winston-Salem, North Carolina; Wake Forest University School of Medicine, Winston Salem, NC; Atrium Health Wake Forest Baptist Medical Center, Winston Salem, North Carolina; Atrium Health Wake Forest Baptist, Winston-Salem, North Carolina; Wake Forest University School of Medicine, Winston Salem, NC; Wake Forest University School of Medicine, Winston Salem, NC; Atrium Health Wake Forest Baptist, Winston-Salem, North Carolina; Atrium Health Wake Forest Health, Winston Salem, North Carolina; Atrium Health Wake Forest Medical Center, Winston Salem, North Carolina; Atrium Wake Forest Baptist Health/ Wake Forest University School of Medicine, Winston-Salem, North Carolina; Atrium Health Wake Forest Baptist, Winston-Salem, North Carolina

## Abstract

**Background:**

On Jan 31, 2023, ABNA replaced universal mupirocin nasal decolonization in pts admitted to the burn service. The purpose of this study was to evaluate the impact of universal nasal decolonization with ABNA vs mupirocin on infection rates and antimicrobial utilization in this pt population.

**Table 1.**
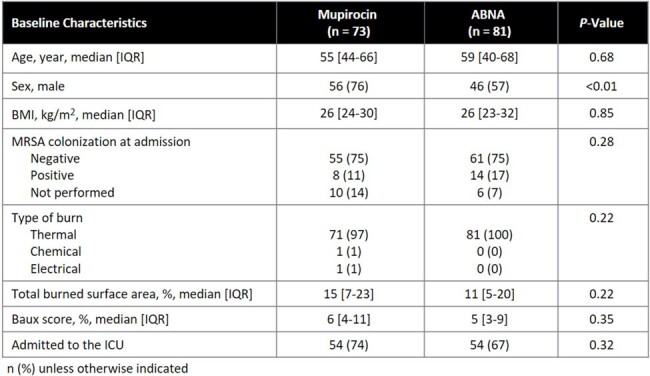
Baseline Characteristics

**Methods:**

This was a single-center, retrospective, cohort analysis of adults ≥ 18 yrs of age admitted to an academic medical center burn service from Feb 2022 - Sept 2022 (mupirocin cohort) and Feb 2023 - Sept 2023 (ABNA cohort) who received ≥ 1 dose of nasal mupirocin or ABNA. Pts were excluded if they were admitted for a non-burn injury, on the burn service for < 4 days, or received nasal decolonization ≤ 6 months prior to admission. The primary outcome was incidence of staphylococcal infection, defined as a staphylococcal organism identified from a blood, respiratory, or wound culture with corresponding diagnosis of pneumonia (PNA), bacteremia, or skin and soft tissue infection (SSTI). Secondary outcomes included incidence of PNA, bacteremia, and SSTIs; MRSA bacteremia LabID event rate and CLABSI event rate as defined by the National Healthcare Safety Network; antimicrobial days of therapy; ICU and hospital length of stay; and hospital mortality.

**Table 2.**
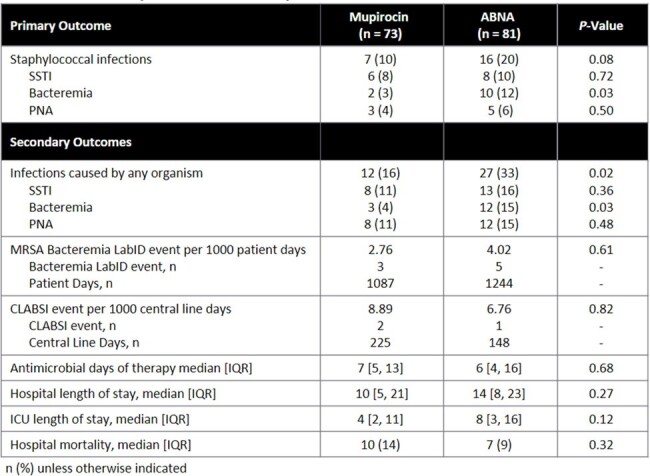
Primary and Secondary Outcomes

**Results:**

73 pts in the mupirocin cohort and 81 pts in the ABNA cohort were included. The only significant difference in baseline characteristics (Table 1) was 56 (76%) pts in the mupirocin cohort were male vs 46 (57%) pts in the ABNA cohort (P < 0.01). Staphylococcal infection occurred in 7 (10%) pts in the mupirocin cohort vs 16 (20%) pts in the ABNA cohort (P = 0.08, Table 2). A Kaplan Meier analysis of time to staphylococcal infection is reported in Figure 1. Secondary outcomes are reported in Table 2. 2 (3%) pts in mupirocin cohort had staphylococcal bacteremia vs 10 (12%) pts in the ABNA cohort (P = 0.03). 12 (16%) pts in the mupirocin cohort vs 27 (33%) pts in the ABNA cohort had an infection (PNA, SSTI, or bacteremia) caused by any organism (P = 0.02), including 3 (4%) pts vs 12 (15%) pts with bacteremia (P = 0.03, Table 3).

**Table 3:**
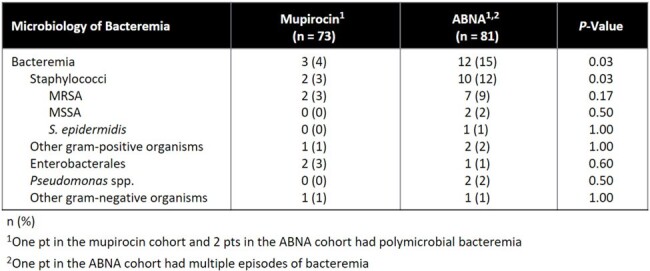
Microbiology of Bacteremia

**Conclusion:**

Pts who received ABNA had a higher incidence of staphylococcal bacteremia, overall bacteremia, and overall infections when compared to pts who received nasal mupirocin. Additional studies are needed to further evaluate the effectiveness of ABNA.

**Figure 1.**
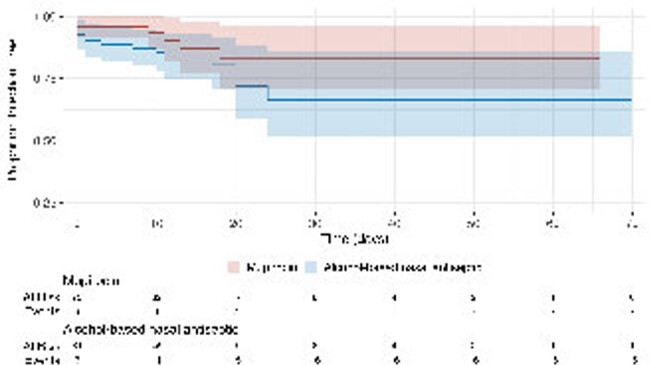
Kaplan Meier Analysis, Time to Staphylococcal Infection

**Disclosures:**

**John C. Williamson, PharmD**, Armata Pharmaceuticals: Grant/Research Support|Blue Collar Vaccines and Therapeutics: Board Member|Blue Collar Vaccines and Therapeutics: Ownership Interest|Paratek Pharmaceuticals: Grant/Research Support|ST Pharm Co, Ltd: Grant/Research Support

